# Low back pain in beauty salons professionals in the city of Fortaleza-CE

**DOI:** 10.47626/1679-4435-2023-1064

**Published:** 2024-09-24

**Authors:** Paulo Ricardo Silva Lopes, Giulianna de Brito Brasil, Thiago Brasileiro de Vasconcelos

**Affiliations:** School of Physical Therapy, Centro Universitário Uninassau Parangaba, Fortaleza, CE, Brazil

**Keywords:** low back pain, work occupation, beauty and aesthetics centers, cumulative trauma disorders, dor lombar, ocupação laboral, centros de embelezamento e estética, transtornos traumáticos cumulativos

## Abstract

**Introduction:**

Low back pain can be defined as pain below the ribs and above the upper gluteal line.

**Objectives:**

The study aimed to analyze low back pain in professionals from beauty salons in the city of Fortaleza, state of Ceará.

**Methods:**

Descriptive, quantitative-qualitative, transversal, non-probabilistic research in the snowball modality, conducted between June and August 2021 in the José Walter neighborhood. Two sociodemographic questionnaires and the Quebec Back Pain Disability scale were applied, which seeks to assess how pain affects the participants’ daily lives.

**Results:**

Forty-two professionals were interviewed, of which 32 women (76.2%), with a mean age of 39.45 ± 10.99 years. Women were more likely to have an onset of low back pain and to live with pain for a longer time compared to men, in addition to these professionals having a significant overload for the hours worked. 52% of respondents showed significant clinical changes, mainly in relation to stand up for 20-30 minutes (16.7%), sit in a chair for several hours (14.3%), walk several kilometers (19%), carry two bags with groceries (14.3%) and lift and carry a heavy suitcase (28.6%).

**Conclusions:**

It was evidenced that low back pain may be related to personal or environmental factors, with a sedentary lifestyle, length of service and working hours as strong indications for the onset of low back pain, with impairment in daily tasks.

## Abstract (pt)

**Introdução:**

A dor lombar pode ser definida como uma dor abaixo das costelas e acima da linha glútea superior.

**Objetivos:**

Analisar a dor lombar em profissionais de salões de beleza na cidade de Fortaleza, estado do Ceará.

**Métodos:**

Tratou-se de pesquisa descritiva, qualiquantitativa, transversal, não probabilística na modalidade bola de neve, realizada entre os meses de junho e agosto de 2021 no bairro José Walter. Foram aplicados dois questionários sociodemográficos e a escala de Quebec Back Pain Disability, que busca avaliar como a dor afeta a vida diária dos participantes.

**Resultados:**

Quarenta e dois profissionais foram entrevistados, sendo 32 mulheres (76,2%), com média de idade de 39,45±10,99 anos. O sexo feminino demonstrou ter maior predisposição para o aparecimento da dor lombar, convivendo com a dor por mais tempo em relação aos homens, além dessas profissionais apresentarem uma sobrecarga significativa para as horas trabalhadas. Dos entrevistados, 52% apresentaram mudanças clínicas significativas, principalmente em relação a ficar em pé por 20 a 30 minutos (16,7%), sentar-se em uma cadeira por várias horas (14,3%), caminhar vários quilômetros (19%), carregar duas sacolas de compras (14,3%) e levantar e carregar uma mala pesada (28,6%).

**Conclusões:**

Evidenciou-se que a dor lombar pode estar relacionada a fatores pessoais ou ambientais, sendo o sedentarismo, o tempo de profissão e as horas trabalhadas fortes indícios para o aparecimento da dor lombar, com comprometimento das tarefas diárias.

## INTRODUCTION

Low back pain (LBP) can be defined as pain below the ribs and above the upper gluteal line, and is classified as the second most common pain complaint worldwide.^1^ Generally, nonspecific LBP can be characterized as muscle tension or stiffness that radiates down to the lower gluteal folds, with pain radiating to the legs or not.^2^

In Brazil, LBP is found to be very common and is highly prevalent. However, the relationship with age has shown that it is more prevalent in 50% of adults and in up to 19.5% of adolescents. It should be noted that chronic LBP is one of the most common disorders affecting the Brazilian population, with an average prevalence of 14.7%.^3^ Machado^4^ observed in his study with hairdressers that lifting loads, inappropriate postures, and no rest are the main risks for musculoskeletal injuries.

In this sense, beauty salon professionals are polyvalent, capable of performing several functions at the same time, usually working as part of a team, for long hours, and in uncomfortable positions. Their working materials include brushes, sandpapers, hair clips, hair dryers, scissors, towels, among others.^5^ Therefore, this study sought to assess the incidence of LBP among beauty salon professionals in the city of Fortaleza, Ceará, Brazil.

## METHODS

This is a descriptive, qualitative-quantitative, cross-sectional, nonprobabilistic snowball study. It was conducted in several beauty salons in the José Walter neighborhood, Fortaleza, Ceará, Brazil, and the first beauty salon interviewed was Lilian Espaço Conceito. The project was approved by the Research Ethics Committee of the Centro Universitário Maurício de Nassau, with Certificate of Presentation for Ethical Appraisal number 52783521.4.0000.5193.

The sample was a nonprobabilistic snowball sample, in which interviews are terminated when data saturation is observed. Baldin & Munhoz^6^ pointed out that the snowball technique is widely used in social research, in which an initial participant indicates new participants and so on until the proposed objective is reached. When the objective is reached and new responses are repetitions of responses already obtained, the saturation point is indicated. The research was carried out between June and August 2021 after signing the Informed Consent Form.

Professionals who met the following inclusion criteria were included in the interviews: residents of the neighborhood surveyed, people of both sexes who work as hairdressers, barbers, or manicurists/pedicurists, aged 18 or over, no mental or neurological dysfunctions, who have been working in the profession for at least 2 years and who have LBP. The exclusion criteria were professionals who were not working in the salons at the time of the survey, off work due to various illnesses (viral, bacterial, etc.) during the interview, and those unwilling to participate in the survey.

Data were collected in person through interviews and two questionnaires: sociodemographic and the Quebec Back Pain Disability Scale (QBPDS),^7,8^ which aims to assess how pain affects participants’ daily lives.

The sociodemographic questionnaire included questions about sex, occupation, marital status, height, body mass index (BMI), educational background, length of service in the profession, length of LBP, whether the pain disappears after the working day, among others.

The Quebec questionnaire was created by Kopec et al.^9^ and validated in Brazil by Rodrigues.^10^ It has a table in which the respondents are requested to mark whether: not difficult at all (score 0), minimally difficult (score 1), somewhat difficult (score 2), fairly difficult (score 3), very difficult (score 4), or whether they are unable to do (score 5), totaling a score of up to 100. If the respondent scores from 0 to 15, they are considered to have no clinical change, and from 16 to 100, they are considered to have some clinical change. Examples of questions included: get out of bed, sleep through the night, ride in a car, walk, run, put on socks, among others.

The data were tabulated on Microsoft Excel and exported to the Statistical Package for the Social Sciences (SPSS) version 20.0 for Windows, where absolute and percentage frequencies were expressed for each piece of data. A p-value < 0.05 was considered statistically significant.

## RESULTS

A total of 42 professionals were interviewed (32 women [76.2%] and 10 men [23.8%]), with a mean age of 39.45±10.99 years and a mean BMI of 27.13±4.42 (overweight). Although most of the sample had no children, those who had given birth reported having a Cesarean section (14 [73.7%]). When asked about their profession, 15 (35.7%) said they were hairdressers (Table 1). All respondents had LBP.

**Table 1:** Sociodemographic data of the interviewed professionals, Fortaleza, CE, Brazil, 2021

Variables	n	%	P
Sex			< 0,01*
Female	32	76.2	
Male	10	23.8	
Do you have any children?			0,22**
Yes	19	45.2	
No	23	54.8	
How many children?			< 0,01**
None	23	54.8	
1	6	14.3	
2	10	23.8	
3	3	7.1	
Profession			0,37**
Hairdresser	15	35.7	
Hairdresser and manicurist/pedicurist	7	16.7	
Manicurist/pedicurist	10	23.8	
Barber	10	23.8	
Educational background			< 0,01**
Some elementary school	2	4.8	
Elementary school	2	4.8	
Some high school	4	9.5	
High school	24	57.1	
Some college degree	5	11.9	
College degree	5	11.9	
Physical exercise			0,87*
Yes	20	47.6	
No	22	52.4	

P = p-value. * Binominal test; * Chi-square test

Table 2 shows the factors that cause pain onset in the respondents (age, weight, BMI, etc.), which were divided according to their job. Grade I and II obesity was mainly present in the manicurists/pedicurists (30%) and barbers (40%).

**Table 2:** Profession and pain aggravating factors, Fortaleza, CE, Brazil, 2021

	Hairdresser	Hairdresser and manicurist/pedicurist	Manicurist/pedicurist	Barber
Age (years)	43.7±9.9	41±12.1	39.0±12.4	31.1 ±7.3
Weight (kg)	66.0±9.5	68.8±11.4	72.8±12.8	82.1 ±17.4
BMI (kg/m^2^)	25.8±3.3	26.6±3.7	28.5±4.6	28.0±6.0
Length of service (years)	17.0±7.3	15.8±9.3	17.0 ±10.4	8.1±6.5
Hours in the same position	3.8±0.3	3.6±0.5	3.7±0.7	4.0±0.1
Length of LBP (years)	6.5±5.4	7.6 ±5.4	7.0±4.9	3.3±2.8

Data expressed as mean and standard deviation. Body mass index (BMI) is calculated by dividing the weight (in kg) by the height squared (in meters); normal = 18.5-24.9; overweight = 25-29.9; grade I obesity = 30-34.9; grade II obesity = 35-39.9.

As for the onset of LBP after starting the working day, men reported pain onset after 4 hours and 30 minutes, while women reported pain onset earlier (3 hours and 5 minutes). As for when the pain disappeared, men reported that the pain disappeared in about 1 hour and 25 minutes and women in about 2 hours and 30 minutes. When asked about pain during the work shift, 24 (57.1%) participants said they had pain at night, compared to the afternoon (11 [26.2%]) and the morning (7 [16.7%]).

Of the 42 participants, 83.3% (35) worked 5 or more hours a day, 14.3% (6) reported working up to 5 hours a day and 2.4% (1) worked 4 hours a day.

Chart 1 shows the correlation between sex, length of service, length of LBP, and physical exercise. A strong correlation was found between sex and occupation (r = 0.77), although a moderate correlation was found between length of service and sex (r = −0.47) and between length of service and length of LBP (r = 0.58).

**Chart 1:** Correlation between variables, Fortaleza, CE, Brazil, 2021

	Sex	Length of service (years)	Age
Occupation			
Spearman’s correlation	0.77	−0.39	−0.41
p-value	0.00*	0.01*	0.00*
Sex			
Spearman’s correlation	1.00	−0.47	−0.39
p-value		0.00*	0.01*
Length of LBP			
Spearman’s correlation	−0.35	0.58	0.50
p-value	0.02*	0.00*	0.00*
Physical exercise			
Spearman’s correlation	0.08	−0.16	0.04
p-value	0.59	0.31	0.76
Age			
Spearman’s correlation	−0.39	0.77	1.00
p-value	0.01*	0.00*	

*Statistically significant.

Only 40.5% (17) of respondents sought out healthcare personnel to treat their pain. The most commonly sought professional was a doctor, with 21.4% (9), compared to a physical therapist, with 9.5% (4).

Table 3 shows the answers to the QBPDS questionnaire, which involves everyday situations and how difficult it is to perform them. The questionnaire has 20 questions, and it is important to highlight 2 answers in score 2 (somewhat difficult): sit in a chair for several hours (33.3%) and lift and carry a heavy suitcase (31%); and 1 answer in score 4 (very difficult): lift and carry a heavy suitcase (28.6%). In addition, 3 questions had answers in score 5 (unable to do): walk a few blocks (300-400 m) (2.4%), walk several kilometers (7.1%) and run one block (about 100 m) (9.5%).

**Table 3:** Answers to Quebec Back Pain Disability Scale (QBPDS) (%), Fortaleza, CE, Brazil, 2021

Variables	0	1	2	3	4	5	P
Get out of bed	64.3	9.5	16.3	7.1	2.4	-	**< 0,01**
Sleep through the night	40.5	26.2	9.5	11.9	11.9	-	**< 0,01**
Turn over in bed	38.1	28.6	21.4	4.8	7.1	-	**< 0,01**
Ride in a car	69	7.1	9.5	9.5	4.8	-	**< 0,01**
Stand up for 20-30 minutes	45.2	19	9.5	9.5	16.7	-	**< 0,01**
Sit in a chair for several hours	35.7	11.9	33.3	4.8	14.3	-	**< 0,01**
Climb one flight of stairs	42.9	23.8	21.4	2.4	9.5	-	**< 0,01**
Walk a few blocks (300-400 m)	47.6	19	14.3	9.5	7.1	2.4	**< 0,01**
Walk several kilometers	38.1	11.9	16.7	7.1	1.9	7.1	**< 0,01**
Reach up to high shelves	76.2	14.3	7.1		2.4		**< 0,01**
Throw a ball	73.8	14.3	2.4	4.8	4.8		**< 0,01**
Run one block (about 100 m)	28.6	31	11.9		19	9.5	0,10
Take food out of the refrigerator	64.3	16.7	7.1	2.4	9.5		**< 0,01**
Make your bed	69	14.3	4.8	2.4	9.5		**< 0,01**
Put on socks (pantyhose)	57.1	23.8	9.5	2.4	7.1		**< 0,01**
Bend over to clean the bathtub	50	21.4	16.7		11.9		**< 0,01**
Move a chair	73.8	19	7.1				**< 0,01**
Pull or push heavy doors	69	21.4		9.5			**< 0,01**
Carry two bags of groceries	35.7	26.2	14.3	9.5	14.3		**< 0,01**
Lift and carry a heavy suitcase	16.7	16.7	31	7.1	28.6		0,09

0 = Not difficult at all; 1 = Minimally difficult; 2 = Somewhat difficult; 3 = Fairly difficult; 4 = Very difficult; 5 = Unable to do. P = p-value by Chi-square test.

**Figure 1:**
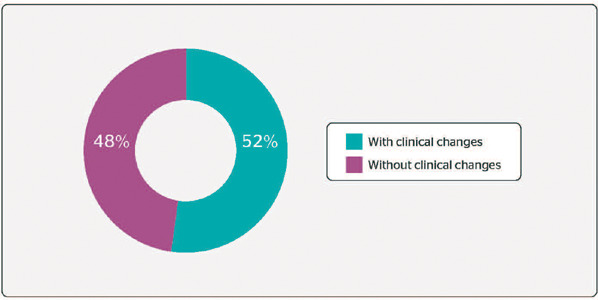
Analysis of the interviews using Quebec Back Pain Disability Scale (QBPDS).

Figure 1 shows the analysis of the interviews with the QBPDS. The scores proposed by Kopec et al.^9^ were used, adding up the points in the 20 questions, which range from 0 to 100 points. We found that 20 professionals (48%) scored below 16 points and thus did not show a clinical change in their daily activities; 22 professionals (52%) scored 16 points or above, showing a clinical change in their daily activities.

## DISCUSSION

All the participants in this study had LBP, which may be related to the posture they have during the working day. This is in line with the findings of the Pesquisa Nacional por Amostra de Domicílios (PNAD, Brazilian National Household Sample Survey) of the Instituto Brasileiro de Geografia e Estatística (IBGE),^11^ which showed that back pain is the second most frequent complaint in Brazil, accounting for 13.5% of the sample.

Participants in this study were mostly women living with LBP. This is due to the higher prevalence of women working in beauty salons than men. It is well known that LBP is a serious health problem worldwide and rates are increasing as the population ages. The Global Burden of Disease (GBD) study^12^ found that LBP was the biggest pain complaint among women in 104 of the 195 territories in which the survey was conducted.

Sedentary lifestyles have been shown to be an important factor in pain. In this study, around 52.4% of the sample did not exercise on a daily basis. Nascimento & Costa^3^ found that LBP is due to various factors (age, sex, income, educational background), and a sedentary lifestyle is an important factor. Foster et al.^13^ showed that the most common treatment for pain, in addition to pharmacological treatment, is gradual physical exercise to improve function and prevent the injury from worsening.

This study sample had all levels of education, but those who had finished high school were the most prevalent. Most of them answered that they did not seek help from health care professionals to deal with their pain, corroborating Akerstrom et al.,^14^ who found that people with a low level of education find it more difficult to treat their pain because they do not seek help from a multidisciplinary team.

With regard to working hours, 83.33% of the sample worked 5 or more hours a day, standing in the same position, revealing that their pain began after 3 hours of work. Medeiros & Medeiros^15^ found that the beauty salon professionals interviewed worked between 6 and 10 hours a day, which can lead to excessive work in the same position, in addition to inappropriate postures that lead to joint pain.

Inadequate postures may be related to the onset of LBP. Oliveira et al.^16^ reported that the workers’ low level of education can affect problems related to posture. In addition, good working conditions are not only related to compliance with government regulations, but also to better working conditions. In this study, workers spent more than 5 hours a day in their workplaces.

Raiser et al.^17^ showed that the workload of professional hairdressers is very stressful. The study included 39 respondents, and 22 reported working more than 10 hours a day. This leads to muscle pain and other occupational illnesses. We can therefore relate this to the results found in this survey, in which 83.3% of respondents reported working 5 hours or more a day.

The Consolidação das Leis do Trabalho (CLT, Brazilian Consolidation of Labor Laws) states that the daily working day should not exceed 8 hours for jobs in any private activity, in order to prevent work-related illnesses. It is therefore important to note that the appearance of LBP may be related to the extra workload of these professionals.^18^

The average BMI in this study was 27.13±4.42 (overweight). This leads to results very similar to those of Souza,^19^ in which 43% of the participants were overweight. Smuck et al.^20^ showed that obese people often develop LBP due to their high BMI. Overweight has not been shown to be a cause of LBP, but when combined with a sedentary lifestyle, overloaded activities, and inadequate postures for a long time, it can lead to the onset of pain.

This study showed that sex and profession were strongly correlated (r = 0.77), showing that women were predominantly involved. This corroborates Geneen et al.,^21^ who found that being a woman is one of the factors that can contribute to the onset of chronic pain, in addition to factors such as age, socioeconomic status, genetics, among others. This study found a moderate relationship between length of service and pain (r = 0.58): the longer the length of service, the greater the pain. Chaise et al.^22^ also pointed out that the older a worker is and the longer they have worked, the more likely they are to develop musculoskeletal pain.

The QBPDS was therefore used as a second source of evaluation. For most of the questions, it was shown that the respondents had no problems doing their activities. However, questions such as sitting in a chair for several hours (33.3% in score 2) and lifting and carrying a heavy suitcase (31% in score 2 and 28.6% in score 4) had the highest percentages for failure. This is consistent with Marques,^7^ who assessed this questionnaire and the same questions had the highest rates: sitting in a chair for several hours had 35.4% in score 3 and carrying a heavy suitcase had 22.6% in score 4 and 6.4% in score 5.

The most relevant response to the degree of difficulty found in the QBPDS is related to sitting in a chair for several hours. We can cite Helfenstein Junior et al.^23^ who reported that sitting for a long time on a daily basis, in the same way as sitting in a flexed position for a long time, can cause fatigue of the extensor muscles of the spine and compromise their stability, leading to increased disability.

As for the QBPDS scores, 52% of the sample had answers equal to or above 16 points. These people fit into the group of clinical changes found with the questionnaire, as seen in Falavigna et al.,^24^ who explain that a score below 15 does not show any clinical change in disability and a score equal to or above 16 is considered a clinical change.

Most of the respondents were sedentary and LBP may be related to poor posture. For this reason, ergonomic interventions and occupational gymnastics need to be implemented for the public surveyed. Soares et al.^25^ pointed out that introducing these activities can prevent or contribute to controlling musculoskeletal symptoms, reducing sick leave and exposure to risk factors. In addition, occupational gymnastics helps workers to have a better perception of their bodies, causing a feeling of well-being and improving their posture.

## CONCLUSIONS

LBP can be related to personal or environmental factors, with sedentary lifestyles, length of service and hours worked being strong indicators of the onset of LBP, with impairment in daily tasks.
